# The variants in PTPRB, TRAF3IP3, and DISC1 genes were associated with Graves’ disease in the Chinese population

**DOI:** 10.1097/MD.0000000000031501

**Published:** 2022-11-11

**Authors:** Wei Li, Haidong Jiang, Xu Chen, Kevin Yang, Xindan Deng, Zheng Tang, Zhihui Hu, Xiaodan Zhang, Shihan Lin, Yuanlin Zou, Hui Wu

**Affiliations:** a Huizhou Health Sciences Polytechnic, Huizhou, China; b Department of Endocrinology, Affiliated Hospital of Guangdong Medical University, Zhanjiang, China; c Department of Cardiology, Sun Yat-sen University, Guangzhou, China.

**Keywords:** genetic polymorphism, graves’ disease, susceptibility genes

## Abstract

Previously, a case series study was conducted on our part in which 5 patients with Graves’ disease (GD) were collected from a 3-generation family to screen for susceptibility genes responsible for GD. The single nucleotide variants of Microtubule-associated protein 7 domain containing 2 c. 452C > T, p. Ala151Val, Solute carrier family 1 member 7 c. 1204C > T, p. Arg402Cys, tumor necrosis factor receptor-associated factor 3 interacting protein 3 (TRAF3IP3) c. 209A > T, p. Asn70Ile, protein tyrosine phosphatase receptor type B (PTPRB) c. 3472A > G, p. Ser1158Gly, Phosphoinositide-3-kinase regulatory subunit 3 c. 121C > T, p. Pro41Ser, disrupted in schizophrenia 1 (DISC1), c. 1591G > C p. Gly531Arg were associated with the familial GD. We then further confirmed these variants and investigated whether other mutations render susceptibility to GD. The case-control study collected patients with sporadic GD or no GD family history. A snapshot program was used for genotyping the selected SNPs in 235 GD patients (GD group 1) and 284 healthy patients (control group). Furthermore, another 184 GD patients were recruited (GD group 2) to sequence the specified exons of these genes. The sequenced data was compared with Chinese Millionome Database (CMDB). Several variants of PTPRB, phosphoinositide-3-kinase regulatory subunit 3, TRAF3IP3, and DISC1 were found in GD group 2 but not in CMDB. Moreover, the allele frequency of SNP rs2076150 (TRAF3IP3) and rs2492367 DISC1 in GD group 2 was significantly higher than that of in CMDB (all *P* < .05). When the control group or CMDB was set as a reference group, a significantly higher frequency in alter allele *C* of SNP rs186466118 PTPRB was observed in GD group 1 and GD group (constituted by GD group 1 and GD group 2). Equally importantly, there was a correlation between the allele C of SNP rs186466118 and the increased risk of GD susceptibility (all *P* < .05). PTPRB, TRAF3IP3, and DISC1 may be susceptibility genes for GD, and more variants of PTPRB, TRAF3IP3, and DISC1 were found in GD patients.

## 1. Introduction

Graves’ disease (GD) is one of the common autoimmune thyroid diseases and multiple genetic polymorphisms, and environmental factors contribute to patients’ susceptibility to GD.^[1]^ Furthermore, documentation of the familial clustering of GD indicated that GD had a heritable risk.^[2,3]^ The main methodological approaches for studying genetic predisposition to GD are based on linkage analysis, candidate genes association studies, and genome-wide association studies. Through genome-wide association studies, the collection of several independent cohorts has facilitated a more thorough screening of candidate gene regions (e.g., human leukocyfe antigen, cytotoxic T lymphocyte-associated antigen-4), fine-mapping GD susceptibility locus remains an unknown realm (such as 6q27, 4p14) and genetic susceptibility regional shared by different autoimmune diseases (e.g., basic leucine zipper transcription factor 2, integrin alpha-M).^[4]^ Previous research has been done in identifying genetic causes of GD, and it was estimated that all known genetic polymorphisms could only explain about 20% of GD heritability. Furthermore, the current GD susceptibility gene study is a distributed case-control study. Hence, it has limited genetic efficacy for interpreting and explaining family aggregation. There is speculation that genetic variation with minor frequency and high-episode variation can cause Graves’ disease familial aggregation.

Our previous work involved 3-generation familial GD patients with a solid genetic background in a Chinese Han population, single nucleotide variants of microtubule-associated protein 7 domain containing 2 (MAP7D2) c. 452C > T, p. Ala151Val, solute carrier family 1 member 7 (SLC1A7) c. 1204C > T, p. Arg402Cys, tumor necrosis factor receptor-associated factor 3 interacting protein 3 (TRAF3IP3) c. 209A > T, (p. Asn70Ile), Protein tyrosine phosphatase receptor type B (PTPRB) c. 3472A > G, p. Ser1158Gly, phosphoinositide-3-kinase regulatory subunit 3 (PIK3R3) c. 121C > T, p. Pro41Ser, disrupted in schizophrenia 1 (DISC1) c. 1591G > C, p. Gly531Arg be associated with the familial GD. Furthermore, the PolyPhen-2 score showed that the variants in TRAF3IP3, PTPRB, and PIK3R3 were more likely to trigger changes in protein functions.^[5]^ In this research, patients with sporadic GD or no GD family history would be collected, and sequencing would be performed on an exon region where the genetic mutation site is located to investigate the relationship between these genetic mutations and sporadic GD patients in the Chinese Han population.

## 2. Methods

### 2.1. Study participants

All the participants were from Guangdong province, China. The inclusion criteria of GD patients and healthy volunteers the American Thyroid Association guidelines. The clinical diagnosis criteria for scientific Graves’ disease in the American Thyroid Association guidelines were the following^[6]^: The etiology of thyrotoxicosis should be determined. Suppose the diagnosis is not apparent based on the clinical presentation and initial biochemical evaluation. In that case, diagnostic testing is indicated. It can include, depending on available expertise and resources; measurement of thyrotrophin receptor antibody; determination of radioactive iodine uptake, or; measurement of thyroidal blood flow on ultrasonography. A^123^I or ^99m^Tc pertechnetate scan should be obtained when the clinical presentation suggests a toxic adenoma or toxic multinodular goiter. The health control group followed the hospital medical examination center and its standards included: there is no abnormality in the case of medical history, medical examination, blood sugar check, blood pressure, blood lipids, and other biochemical examinations; the subject, and the 3 generations of family members are not associated with autoimmune diseases in GD. Furthermore, healthy volunteers were excluded from those with a family history of autoimmune disease, including GD. GD group 1 was used to determine the association between GD susceptibility and mutations. GD group 2 was used to screen GD-susceptible related mutations in the selected genes’ exon region. The general characteristics of the study population are described in Table 1. The Ethics Committee approved the research of the Affiliated Hospital of Guangdong Medical University (Approval NO. PJ2012029) and the informed consent form of all participants.

**Table 1:** The general characteristics of study population.

	GD group 1	GD group 2	Control group	*P* value
Sex(male/female)	83/154	57/128	110/175	0.897
Age(year)	37.33 ± 13.01	37.36 ± 14.57	36.84 ± 14.93	0.186

Data are mean ± standard deviation (SD) or *n*, GD = Graves’ disease.

### 2.2. Investigate the potential mutations that render susceptibility to GD

**Figure 1.**
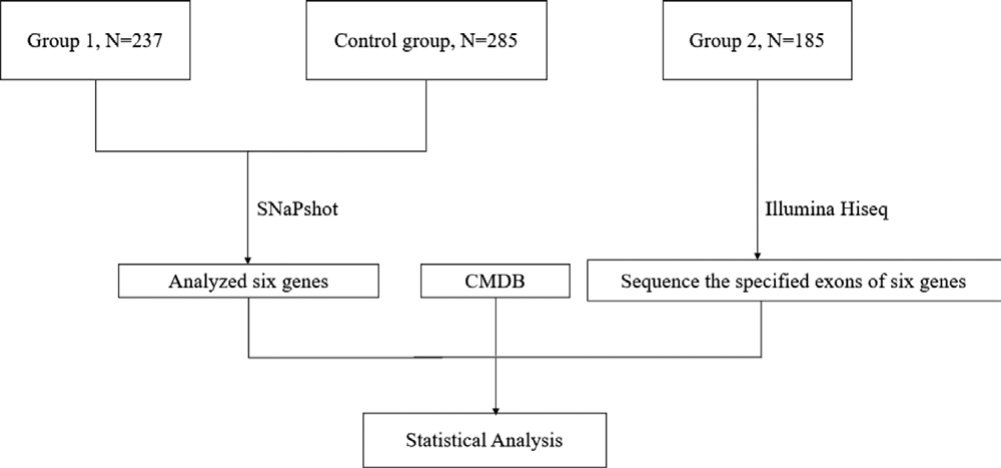
The brief flow chart of the experiment. CMDB = Chinese Millionome Database.

After the 3 generations’ variants in the familial GD of 3 generations were obtained, an unrelated population was composed of 237 GD patients (GD group 1) and 285 healthy controls. For the preparation of DNA, genomic DNA from EDTA-treated peripheral blood was extracted according to the DNA extraction kit manual (Tiangen Biochemical Technology Co., Ltd.). The selected variants of GD patients and control subjects were analyzed through Snapshot (Life Technologies, USA). There was further research on SNPs in the specified exons of PIK3R3 (exon 2), PTPRB (exon 14), MAP7D2 (exon 4), TRAF3IP3 (exon 3), DISC1 (exon 6), SLC1A7 (exon8) for its contribution to GD susceptibility. Another 184 GD patients (GD group 2) were recruited to sequence these specified exons of genes. Sequencing of the exon was finished through Illumina Hiseq/MiSeq sequencing platform. After obtaining the sequencing data (the detailed information seen in additional files), it was then compared to the Chinese Millionome Database (CMDB, http://cmdb.bgi.com/), which contains considerable variation and allelic frequency information from 141,431 unrelated healthy Chinese individuals (Phase I results). Finally, GD group 1 and GD group 2 were merged into the GD group, then compared to the GD group, control group, and CMDB. The flow chart of the case series study is shown in Figure 1.

### 2.3. Statistical analysis

Empower (R) (www.empowerstats.com, X&Y solutions, Inc., Boston, MA) and R (http://www.R-project.org) were applied to all statistical analyses. Fisher’s exact test determined differences in allelic and genotypic distribution between the GD group (GD group 1 and GD group 2) and the control group (or CMDB). *χ*^2^ tests or *T*-tests were applied to compare the general characteristics of the study population. The *P*-value was less than 0.05 (2-tailed) as statistical significance.

## 3. Results

A summary of a GD case-control study of each SNP is presented in Table 2. There are 2 sample genotyping failures in GD group 1 and 1 sample genotyping failure in the control group. When the control group or CMDB were reference group, there was a significantly higher allelic frequency of PTPRB polymorphism in GD group 1 and GD group (*P*^a^ = 0.019 for GD group 1 vs Control group, *P*^b^ = 0.004 for GD group 1 vs CMBD, *P*^c^ = 0.013 for GD group vs Control group, *P*^d^ = 0.004 for GD group vs CMBD). The allele *C* of SNP rs186466118 (PTPRB) significantly increased the risk of GD susceptibility. The allele A frequency of SNP rs115181807 was significantly higher in GD group 1 than in the control group but could not be found in other comparisons. There was no difference in the other 4 variants’ frequency in all the comparisons (all *P* > .05).

**Table 2:** Analyzing the association between GD and variants.

	Alt/Ref allele count (N)					
	MAP7D2	PIK3R3	SLC1A7	TRAF3IP3	DISC1	PTPRB
Control group	0/568	0/568	0/568	1/567	13/555	0/568
CMDB	-	77/ 14,962	-	-	-	36/ 9275
GD group 1	0/470	5/465	0/470	1/469	12/458	6/464
GD group	0/838	6/832	-	1/837	24/814	9/829
*P* ^a^ value	NA	0.019	NA	1	0.84	0.008
*OR*^a^ (95%*CI*)	NA	2.22 (2.08-2.38)	NA	1.1 (0.28-4.42)	1.06 (0.70-1.61)	2.22 (2.08-2.38)
*P*^b^ value	NA	0.104	NA	NA	NA	0.004
*OR*^b^(95%*CI*)	NA	2.09 (0.84-5.19)	NA	NA	NA	3 (1.42-6.32)
*P*^c^ value	NA	0.087	NA	1	0.51	0.013
*OR*^c^ (95%*CI*)	NA	1.68 (1.61-1.76)	NA	0.68 (0.04-10.85)	1.26 (0.64-2.49)	1.69 (1.61-1.76)
*P*^d^ value	NA	0.45	NA	NA	NA	0.004
*OR*^d^ (95%*CI*)	NA	1.37 (0.63-3.00)	NA	NA	NA	2.44 (1.35-4.40)

CMDB = Chinese Millionome Database, DISC1 = Disrupted in schizophrenia 1, MAP7D2 = Microtubule-associated protein 7 domain containing 2, PIK3R3 = Phosphoinositide-3-kinase regulatory subunit 3, PTPRB = Protein tyrosine phosphatase receptor type B, SLC1A7 = Solute carrier family 1 member 7, TRAF3IP3 = Tumor necrosis factor receptor-associated factor 3 interacting protein 3; Pa, ORa: GD group 1 VS. Control group; Pb, ORb: GD group 1 VS. CMBD; Pc, ORc: GD group VS. Control group; Pd, ORd: GD group VS. CMBD, GD = Graves’ disease.

Sequencing results for specified exons were shown in Table 3. When all the sequencing results were compared with CMDB, PTPRB variants were found (G ＞ A in chr12:70956826 position, C > T in chr12:70956870 position), PIK3R3 variants were found (T> C in chr1:46546377 position), TRAF3IP3 variants were found (A> T in chr1: 209933593, rs2076151), and DISC1 variants were found (rs56229136) genes in GD group 2. Nevertheless, these variants were not found in CMDB. Of these variants, all except for SNP rs2076151 were rare frequently. The altered allele frequency of SNP rs2076150 and rs2492367 were significantly higher than that in CMDB (all *P* < .05) but was not observed in SNP rs115181807, rs186466118, and rs2076149. There were no variants in the specified exon of MAP7D2 and SLC1A7.

**Table 3:** Sequencing of selected exons in GD group 2 and analyzing the association between GD group 2 and CMDB.

Rs id	Alleles change	Position	Alt/Ref allele count (N)		*P* value	*OR* (95%CI)
			GD group2	CMDB		
**PIK3R3**						
-	T>C	chr1:46546377	1/367	-	NA	NA
rs115181807	G>A	chr1:46546408	1/365	77/ 14,962	0.80	0.54(0.07-3.86)
**PTPRB**						
rs186466118	T>C	chr12:70956666	3/365	36/ 9275	0.26	2.12(0.65-6.91)
-	G>A	chr12:70956826	1/369	-	NA	NA
-	C>T	chr12:70956870	1/369	-	NA	NA
**TRAF3IP3**						
rs2076149	G>A	chr1:209933528	111/259	1941/4952	0.44	1.09(0.87-1.37)
rs2076150	A>G	chr1:209933540	128/240	1249/ 5308	<0.001	2.27(2.24-2.83)
-	A>T	chr1:209933593	2/366	-	NA	NA
rs2076151	G>C	chr1:209933660	67/301	-	NA	NA
**DISC1**						
rs2492367	C>T	chr1:231906589	66/302	1020/ 7208	0.002	1.54(1.17-2.03)
rs56229136	G>C	chr1:231906773	12/356	-	NA	NA
**MAP7D2**	-	-	-	-	-	-
**SLC1A7**	-	-	-	-	-	-

CMDB = Chinese Millionome Database, DISC1 = Disrupted in schizophrenia 1, GD = Graves’ disease, MAP7D2 = Microtubule-associated protein 7 domain containing 2, PIK3R3 = Phosphoinositide-3-kinase regulatory subunit 3, PTPRB = Protein tyrosine phosphatase receptor type B, SLC1A7 = Solute carrier family 1 member 7, TRAF3IP3 = Tumor necrosis factor receptor-associated factor 3 interacting protein 3.

## 4. Discussion

There is an implication of genetic susceptibility in familial GD than sporadic GD. The region of 5q31-q33, 6p, 7q, 8q, 10q, 12q, 14q, 20q and the human leukocyfe antigen, CTLA-4, and TSHR genes were studied in previous familial GD susceptibility studies.^[7–12]^ However, the conclusions were not consistent among different human races. Only 2 definite genes in 3-generation familial GD were reported in the Chinese population, but they weren’t examined. The TSHR germline mutation (ATG → GTG; Met463Val) was identified in a 3-generation family with 8 hyperthyroid members.^[13]^ In a previous study, polymorphisms of MAP7D2, SLC1A7, TRAF3IP3, PTPRB, PIK3R3, and DISC1 were found in familial GD. There was a hypothesis that the mutations in PTPRB, PIK3R3, and TRAF3IP3 might alter the encoded protein functions.^[5]^ To further confirm the relevance of the above polymorphisms and GD susceptibility, sporadic GD patients were recruited for further analysis, and mutations related to GD susceptibility were sequenced. In subsequent experiments, several variants were found in PTPRB (G>A in chr12:70956826 position, C>T in chr12:70956870 position), PIK3R3 (T>C in chr1: 46546377 positions), TRAF3IP3 (A>T in chr1: 209933593, rs2076151), DISC1 (rs56229136) genes in GD group 2. However, no variants were found in CMDB. The altered allele frequency of SNP rs2076150 (TRAF3IP3) and rs2492367 DISC1 was significantly higher than in CMDB. When the control group or CMDB were set as referenced groups, there was a significant increase in altered allele C of SNP rs186466118 (PTPRB) in GD group 1 and GD group. The allele C of SNP rs186466118 also increased the risk of GD susceptibility significantly. Of these variants, some alleles count and frequency were relatively less in our sample, so higher sample size cohorts are warranted to confirm the findings. There were no details about the family history of the GD group, which restricts further understanding of the role of these variants.

Both PTPRB and TRAF3IP3 participated in innate immune maturation. The protein encoded by PTPRB belongs to the family (PTP) of the protein tyrosine phosphatase (PTP), and the protein is involved in the regulation of tyrosine phosphatase. The protein tyrosine phosphate is involved in the immune response. Studies have demonstrated that tyrosine phosphate-dependent signaling pathways are essential for NK and neutrophil cells.^[14,15]^ TRAF3IP3 has been highly expressed in typical lymphatic progenitor cells and CD34 + CD38 + CD7 + cells which can differentiate into B and T cells, suggesting that Traf3IP3 can play in lymphatic development.^[16]^ Moreover, TRAF3IP3 contributed to B lymphocyte-related autophagy and *T* cell development.^[17,18]^ Equally importantly, multi-targeted receptor tyrosine kinase inhibitors used for carcinoma induce thyroid dysfunction.^[19,20]^ Most of these receptor tyrosine kinase inhibitors could reduce the thyroid hormone level and increase the TSH concentration.^[20,21]^ In addition to *T* cell superactivation, recent autophagy has been shown to play a role in thyroid-specific autoimmune. Therefore, it was implied that PTPRB and TRAF3IP3 might be essential GD candidate genes. Until now, only the rs2492367 polymorphism in DISC1 has been shown to be associated with the bipolar affective disorder; there were no reports about the other SNPs of PTPRB, TRAF3IP3, and DISC1 genes that can affect protein function and disease. There needs to be further research on these genes.

More variants of PTPRB, TRAF3IP3, and DISC1 were associated with GD susceptibility. The results further confirmed the previous study on the relationship between susceptibility genes and GD. The PTPRB, TRAF3IP3, and DISC1 genetic polymorphisms may play a role in GD susceptibility.

## 5. Conclusion

The results in this case-control study confirmed that PTPRB, TRAF3IP3, and DISC1 might be susceptibility genes for GD. Furthermore, more variants of PTPRB, TRAF3IP3, and DISC1 were found in GD patients.

## Author contributions

**Conceptualization:** Wei Li, Zhihui Hu, Xiaodan Zhang, Hui Wu.

**Data curation:** Wei Li, Haidong Jiang, Zhihui Hu, Xiaodan Zhang, Hui Wu.

**Formal analysis:** Haidong Jiang, Zhihui Hu, Xiaodan Zhang, Hui Wu.

**Funding acquisition:** Kevin Yang.

**Investigation:** Kevin Yang, Zheng Tang.

**Project administration:** Wei Li, Kevin Yang.

**Resources:** Xu Chen, Zheng Tang, Shihan Lin, Yuanlin Zou.

**Software:** Xu Chen, Kevin Yang, Shihan Lin, Yuanlin Zou.

**Supervision:** Xindan Deng, Zheng Tang.

**Visualization:** Xu Chen, Xindan Deng, Zheng Tang, Zhihui Hu.

**Writing** – **original draft:** Xu Chen, Zheng Tang, Xiaodan Zhang, Hui Wu.

**Writing** – **review & editing:** Wei Li, Haidong Jiang, Xiaodan Zhang, Hui Wu
